# Supplementary material for: Comparative Efficacy of the Novel Diarylquinoline TBAJ-587 and Bedaquiline against a Resistant Rv0678 Mutant in a Mouse Model of Tuberculosis
Source: Antimicrob Agents Chemother. 2021 Mar 18;65(4):e02418-20. doi: 10.1128/AAC.02418-20 (PMC8097419; doi:10.1128/AAC.02418-20)
Supplement: Supplemental file 1 [file AAC.02418-20-s0002.pdf]

Supplementary Table 1. Experimental scheme for mice infected with wild-type *M. tuberculosis* H37Rv

Regimen	No. of mice[a]				
	D-13	D0	M1	M2	Total
Untreated	5	5			10
B_{25}			5	5	10
S_{25}			5	5	10
S_{50}			5	5	10
PaL			5		5
PaMZ			5		5
B_{25}PaL			5		5
B_{25}PaMZ			5		5
S_{25}PaL			5		5
S_{25}PaMZ			5		5
S_{50}PaL			5		5
S_{50}PaMZ			5		5
Total	5	5	55	15	80

[a]Time points shown as days (D-13 or D) or months (M1 or M2) of treatment.

Abbreviations:

B=Bedaquiline; Pa=Pretomanid; L=Linezolid, M=moxifloxacin, Z=Pyrazinamide; S= TBAJ-587. Doses are indicated in subscripts.

Supplementary Table 2. Experimental scheme for mice infected with *M. tuberculosis* H37Rv *Rv0678* mutant

Regimen	No. of mice sacrificed[a]				
	D-13	D0	M1	M2	Total
untreated	5	5	5		15
$B_{12.5}$			5		5
B_{25}			5		5
B_{50}			5		5
S_{25}			5		5
S_{50}			5		5
$Pa_{100}L_{100}$			5	5	10
$PaM_{100}Z_{150}$			5	5	10
$B_{25}PaL$			5	5	10
$B_{25}PaMZ$			5	5	10
$S_{25}PaL$			5	5	10
$S_{25}PaMZ$			5	5	10
$S_{50}PaL$			5	5	10
$S_{50}PaMZ$			5	5	10
Total	5	5	70	40	120

[a]Time points shown as days (D-13 or D) or months (M1 or M2) of treatment.

Abbreviations:

B=Bedaquiline; Pa=Pretomanid; L=Linezolid, M=moxifloxacin, Z=Pyrazinamide; S= TBAJ-587. Doses are indicated in subscripts.

Supplementary Table 3. Lung CFU counts assessed during treatment against the *M. tuberculosis* H37Rv strain.

Regimen	**Mean (±SD) log_{10} CFU count at[a]:**			
	D-13	D0	M1	M2
Untreated	4.11±0.06	7.79±0.11		
B_{25}			4.50±0.14	2.87±0.03
S_{25}			2.92±0.14	1.03±0.60
S_{50}			2.71±0.18	0.42±0.57
PaL			6.26±0.05	
B_{25}PaL			3.38±0.52	
S_{25}PaL			1.80±0.50	
S_{50}PaL			1.23±0.18	
PaMZ			3.61±0.23	
B_{25}PaMZ			1.84±0.46	
S_{25}PaMZ			0.40±0.16	
S_{50}PaMZ			0.31±0.43	

[a]Time points shown as days (D-13 or D) or months (M1 or M2) of treatment.

Abbreviations:

B=Bedaquiline; Pa=Pretomanid; L=Linezolid, M=moxifloxacin, Z=Pyrazinamide; S= TBAJ-587. Doses are indicated in subscripts.

Supplementary Table 4. Lung CFU counts assessed during treatment against the *M. tuberculosis* H37Rv strain with an *Rv0678* mutation.

Regimen	**Mean (±SD) $\log_{10}$ CFU count at[a]:**			
	**D-13**	**D0**	**M1**	**M2**
untreated	4.19±0.08	7.75±0.10		
$B_{12.5}$			7.46±0.02	
B_{25}			7.19±0.18	
B_{50}			6.99±0.11	
S_{25}			5.74±0.16	
S_{50}			5.48±0.10	
PaL			6.65±0.21	4.70±0.35
B_{25}PaL			5.53±0.22	3.15±0.36
S_{25}PaL			3.88±0.18	0.82±0.76
S_{50}PaL			3.96±0.30	0.84±0.21
PaMZ			3.57±0.16	0.53±0.56
B_{25}PaMZ			1.84±0.35	0.17±0.23
S_{25}PaMZ			1.20±0.28	0±0
S_{50}PaMZ			0.81±0.20	0±0

[a]Time points shown as days (D-13 or D) or months (M1 or M2) of treatment.

Abbreviations:

B=Bedaquiline; Pa=Pretomanid; L=Linezolid, M=moxifloxacin, Z=Pyrazinamide; S= TBAJ-587. Doses are indicated in subscripts.

Supplementary Table 5. Proportions of mice and $\log_{10}$ counts of *M. tuberculosis* CFU showing resistance to bedaquiline (BDQ, red, 0.06 μg/ml; blue, 0.25 μg/ml) after infection with wild-type *M. tuberculosis* and antimicrobial treatment.

	Proportion of mice with detectable BDQ-resistant subpopulations and mean (±SD) CFU counts on drug-containing plates (mean frequency of resistant CFU among all CFU recovered)*							
	M1					M2		
Drug, dose (mg/kg)	Monotherapy	DARQ combined with PaL		DARQ combined with PaMZ		Monotherapy	DARQ combined with PaL	
	BDQ-resistant	BDQ-resistant	PMD-resistant	BDQ-resistant	PMD-resistant	BDQ-resistant	BDQ-resistant	PMD-resistant
None	NT	5/5, 2/5<br>($8.7x10^{-5}$)<br>2.07±0.41<br>1.66, 1.20	NT	4/5<br>($4.5x10^{-3}$)<br>1.23±0.11	NT	NT	NT	NT
BDQ (25)	5/5<br>($2.9x10^{-2}$)<br>2.57±0.65<br>1.04, 1.32	3/5<br>($8.1x10^{-3}$)<br>1.14±0.62	0/5	1/5<br>($1.8x10^{-1}$)<br>1.61	NT	5/5<br>($1.8\ x10^{-1}$)<br>2.04±0.30<br>1.07±0.34	NT	NT
TBAJ-587 (25)	0/5	0/5	0/5	0/5	NT	1/5<br>($3.3\ x10^{-1}$)<br>0.78	NT	NT
TBAJ-587 (50)	0/5	0/5	0/5	0/5	NT	0/5	NT	NT

Abbreviations:
BDQ=bedaquiline; PMD, Pa=pretomanid; L=linezolid, M=moxifloxacin; Z=pyrazinamide;
M1 and M2, months of treatment; NT=not tested
At Day 0: Mean (±SD) CFU counts on plates containing 0, 0.06 and 0.25 μg/ml BDQ were: 7.79±0.11, 3.35±0.27 and 2.91±0.41 $\log_{10}$ CFU, respectively.
*reported frequency and CFU counts include only mice in which resistant subpopulations were detected

Supplementary Table 6. Proportions of mice and $\log_{10}$ counts of *M. tuberculosis* CFU showing resistance to bedaquiline (BDQ, red, 1 µg/ml) and pretomanid (PMD, green, 2.0 µg/ml) after infection with the *M. tuberculosis Rv0678* mutant and antimicrobial treatment.

	Proportion of mice with detectable BDQ- or PMD-resistant subpopulations and mean (±SD) CFU counts on drug-containing plates (mean frequency of resistant CFU among all CFU recovered)*									
	M1					M2				
Drug, dose (mg/kg)	Mono-therapy	DARQ combined with PaL		DARQ combined with PaMZ		Mono-therapy	DARQ combined with PaL		DARQ combined with PaMZ	
	BDQ-resistant	BDQ-resistant	PMD-resistant	BDQ-resistant	PMD-resistant	BDQ-resistant	BDQ-resistant	PMD-resistant	BDQ-resistant	PMD-resistant
None	NT	2/5<br>(1.4x10^{-6})<br>0.78, 1.04	NT	0/5	NT	NT	0/5	5/5<br>(9.0x10^{-4})<br>1.55±0.63	0/5	0/5
BDQ (12.5)	1/3<br>(1.9x10^{-7})<br>0.78	NT	NT	NT	NT	NT	NT	NT	NT	NT
BDQ (25)	0/5	1/5<br>(2.7x10^{-5})<br>1.20	5/5<br>(1.4x10^{-4})<br>1.31±0.54	0/5	NT	NT	0/5	5/5<br>(6.1x10^{-2})<br>1.52±0.44	0/5	0/5
BDQ (50)	0/5	NT	NT	NT	NT	NT	NT	NT	NT	NT
TBAJ-587 (25)	0/5	0/5	0/5	0/5	NT	NT	0/5	0/5	0/5	0/5
TBAJ-587 (50)	0/5	0/5	1/5<br>(3.6x10^{-3})<br>1.32	0/5	NT	NT	0/5	0/5	0/5	0/5

Abbreviations:
BDQ=bedaquiline; PMD, Pa=pretomanid; L=linezolid, M=moxifloxacin; Z=pyrazinamide; M1 and M2, months of treatment; NT=not tested
*reported frequency and CFU counts include only mice in which resistant subpopulations were detected
